# Editorial: Pathogen transmission at the domestic-wildlife interface: a growing challenge that requires integrated solutions, volume II

**DOI:** 10.3389/fvets.2026.1780843

**Published:** 2026-01-27

**Authors:** Saúl Jiménez-Ruiz, Ferran Jori, Nuno Santos, José A. Barasona, Amanda E. Fine

**Affiliations:** 1 Grupo Sanidad y Biotecnología (SaBio), Instituto de Investigación en Recursos Cinegéticos IREC (UCLM-CSIC-JCCM), Universidad de Castilla-la Mancha, Ciudad Real, Spain; 2 GISAZ Animal Health and Zoonoses Research Group, ENZOEM Zoonoses and Emergent Diseases Competitive Research Unit, Department of Animal Health, University of Córdoba, Córdoba, Spain; 3 UMR ASTRE (Animal, Santé, Territoires, Risque et Ecosystèmes), CIRAD-INRAE, University of Montpellier, Montpellier, France; 4 Department of Zoology and Entomology, University of Pretoria, Pretoria, South Africa; 5 InBIO Associated Laboratory, CIBIO Research Centre in Biodiversity and Genetic Resources, University of Porto, Vairão, Portugal; 6 BIOPOLIS Program in Genomics, Biodiversity and Land Planning, Vairão, Portugal; 7 VISAVET Health Surveillance Centre, Complutense University of Madrid, Madrid, Spain; 8 Department of Animal Health, Faculty of Veterinary Medicine, Complutense University of Madrid, Madrid, Spain; 9 Wildlife Conservation Society, Health Program, New York, NY, United States

**Keywords:** antimicrobial resistance, biosecurity, disease management, domestic-wildlife interface, eco-epidemiology, risk assessment, surveillance

## 1 Introduction

Domestic-wildlife interfaces constitute dynamic and heterogeneous systems in which ecological, epidemiological, and socio-economic processes converge, creating opportunities for pathogen transmission between wildlife, domestic animals, humans, and the shared environment. These interfaces evolve in response to several factors including land-use change, agricultural intensification, wildlife population recovery, animal trade, globalization, or human mobility, among others, all of which reshape pathogen transmission pathways across spatial and temporal scales (1, 2).

Volume I emphasized the need for integrative and interdisciplinary research to characterize eco-epidemiological drivers in these complex epidemiological systems to inform disease management and control strategies (1). Building directly upon this conceptual framework, Volume II continues the reflection around this topic by focusing on applied eco-epidemiology, risk-based surveillance, and intervention-oriented research. Together, both volumes provide a coherent and complementary background that promotes the implementation of One Health approaches at different domestic-wildlife interfaces.

## 2 Organization of the Research Topic and new findings

The eight articles included in Volume II reflect the diversity and complexity of domestic-wildlife interfaces worldwide. While conceptually aligned with Volume I, the thematic structure of this second Research Topic is shaped by the applied nature of the individual contributions and can be grouped into three interconnected thematic axes: (i) eco-epidemiological drivers and pathogen transmission pathways (50.0%; 4/8); (ii) surveillance of antimicrobial resistance (12.5%; 1/8); and (iii) management and intervention strategies (37.5%; 3/8).

### 2.1 Eco-epidemiological drivers and transmission pathways

Rosen et al. critically reassess the long-standing paradigm of veterinary fencing as a disease control measure in the Kavango-Zambezi Transfrontier Conservation Area (TFCA), the largest terrestrial TFCA in the world. Historically erected to prevent transboundary animal diseases such as foot-and-mouth disease, contagious bovine pleuropneumonia, and peste des petits ruminants, these fences have fragmented habitats and disrupted migratory routes for many wild mammals like African elephants (*Loxodonta* spp.). Using a qualitative risk-assessment framework, the authors demonstrate that removing selected fence sections does not increase the risk of disease transmission, particularly when complemented by measures such as strategic herding. They also show that, in other areas, fencing effectiveness is often compromised by landscape heterogeneity, wildlife movement ecology, and practical constraints related to fence maintenance. These findings support a shift toward science-based, integrative solutions for disease prevention and control that reconcile animal health, wildlife conservation, and community wellbeing within a One Health framework, consistent with previous studies in Southern Africa or Mediterranean contexts (3, 4).

Minicucci et al. examine the eco-epidemiology of *Neospora caninum* at the domestic-wildlife interface in Minnesota, where neosporosis remains a major cause of abortion in cattle. They aimed to evaluate both producer and veterinarian perceptions of disease risk, to assess seroprevalence in cattle, domestic animals, and wildlife, and to identify management factors influencing pathogen transmission. By integrating human assessments with ecological and epidemiological data, the authors sought to clarify whether wolves (*Canis lupus*) -often blamed by producers- play a significant role in disease dynamics compared to other hosts. Their results reveal a clear mismatch between perceived and empirically supported transmission risks, particularly regarding the role of wildlife hosts and on-farm management practices. This aligns with a growing body of literature indicating that human perception and behavior are key, yet often under-recognized, drivers of disease dynamics at these interfaces (5, 6). Interestingly, the authors show that incorporating social dimensions into disease risk analyses demonstrates that management decisions are usually taken following perceptions rather than scientific evidence, which may inadvertently shape transmission pathways.

Two complementary studies by Gao et al. further broaden the ecological and taxonomic scope of intestinal parasite transmission networks at the domestic-wildlife interface. In the first one, Gao, Qin, et al. report *Blastocystis* spp. and *Enterocytozoon bieneusi* in raptors, extending the known host range of these parasites to predatory avian species. Although the overall prevalence was low (< 2%), the detection of subtypes ST3 and ST10, both associated with human and livestock *Blastocystis* spp. infections, and *E. bieneusi* genotypes BEB6 and CHN-F1, known for zoonotic potential and waterborne transmission, is highly significant. These findings suggest that raptors may contribute to cross-species transmission and environmental contamination, particularly through fecal deposition in water sources. In the second, Gao, Gao, et al. report a high prevalence (>65%) and notable genetic diversity (identification up to 10 different subtypes) of *Blastocystis* spp. in farmed sika deer (*Cervus nippon*) in northern China. The close genetic similarity between deer-, human- and livestock-derived sequences reinforces the potential for cross-species transmission, particularly in wildlife farming systems where human-animal contact is frequent (6), pointing toward farmed cervids as amplification hosts for enteric pathogens in these scenarios. Raptors and cervids, though ecologically distinct, share the capacity to maintain and disseminate parasites with zoonotic potential, creating transmission pathways that extend beyond species boundaries. Together, these findings support previous observations that pathogens often move through complex food networks and multi-trophic interactions, underscoring the relevance of considering entire ecological networks rather than isolated host pairs at different interfaces (7).

### 2.2 Surveillance of antimicrobial resistance

Valença et al. report the detection of ESBL/pAmpC-producing *Enterobacterales* in captive common leopard geckos (*Eublepharis macularius*) and central bearded dragons (*Pogona vitticeps*) in Portugal. They found a total of 17.9% of reptiles sampled harbored third-generation cephalosporin-resistant strains such as *Citrobacter freundii, Klebsiella aerogenes*, and *Escherichia coli*. Detected resistance genes (*bla*CMY-2, *bla*CTX-M-15, *bla*TEM-1) and multidrug resistance profiles point the role of non-traditional hosts (exotic pets) as reservoirs of antimicrobial resistance in urban settings. These findings align with existing evidence that antimicrobial resistance determinants can circulate across wildlife, domestic animals, and human-managed environments, in this case, facilitated by the exotic pet trade, captive management, and environmental contamination (8). This contribution underscores the need to expand surveillance efforts beyond conventional livestock and wildlife species to capture emerging and unconventional interface scenarios.

### 2.3 Management and intervention strategies

African swine fever (ASF) continues leading to significant economic losses at the swine husbandry worldwide, while disease control in wild boar (*Sus scrofa*) introduces several challenges that complicate eradication efforts. Ibáñez-Porras et al. introduce WiBISS (Wild Boar Immunization Simulation System), a novel modeling tool designed to estimate the economic benefits of hypothetical ASF vaccination campaigns in wild boar populations in Northern Italy at the municipality scale. Although ASF vaccines for wild boar are not yet commercially available in Europe, WiBISS offers a valuable tool for future planning. By integrating epidemiological risk with economic impact assessment, this tool provides a pragmatic framework that helps authorities prioritize resources, surveillance and intervention strategies and anticipate trade-related impacts to guide decision-making, even under limited resources. Its simplicity and adaptability make it particularly relevant for early outbreak response, complementing more complex alternatives. Ultimately, WiBISS exemplifies how innovative modeling approaches can inform One Health strategies, balancing disease control, wildlife management, and economic sustainability in the fight against emerging diseases, such as ASF.

Bovine tuberculosis (bTB) remains a persistent challenge at the domestic-wildlife interface in Michigan (USA), where free-ranging white-tailed deer (*Odocoileus virginianus*) serve as a self-sustaining reservoir of *Mycobacterium bovis*. Despite decades of control efforts based on increased harvest, baiting bans, and farm-level biosecurity, among many others, the apparent prevalence in deer has stalled between 1 and 2%, sustaining spillback transmission to cattle and posing zoonotic risks. VerCauteren et al. present the first field evaluation of oral delivery of a bTB vaccine (Bacillus Calmette-Guérin) to free-ranging deer, a strategy aimed at complementing existing measures, following modeling investigations (9). The authors strengthen the case for incorporating wildlife-targeted interventions into broader bTB control programs, particularly in regions where wildlife reservoirs undermine livestock-focused strategies. Incorporating biomarkers in future trials will enable differentiation between vaccinated and infected animals, addressing diagnostic challenges.

Complementing this intervention-oriented contribution, de Klerk-Lorist et al. report the first documented outbreak of clinical bTB in free-ranging vervet monkeys (*Chlorocebus pygerythrus*) within the Greater Kruger Conservation Area (GKCA), a region where *M. bovis* has long been maintained in African buffalo (*Syncerus caffer*) and spilled over to multiple wildlife and domestic species. Between June 2023 and May 2024, six vervet monkeys with severe bTB clinical signs were either found dead or euthanized in two tourist areas of GKCA. Lesions were extensive, affecting the lungs, spleen, and different lymph nodes. Comprehensive diagnostic work confirmed *M. bovis* infection in all cases. The severity and distribution of lesions indicate possible oral/aerosol transmission routes, while behavioral observations raise concerns about indirect exposure to sympatric humans or other species. Vervet monkeys are highly social and frequently interact with humans in tourist settings, creating opportunities for cross-species transmission. These findings have significant implications for bTB disease surveillance and public health. This case series underscores the complexity of multi-host disease dynamics and the relevance of One Health approaches in high-contact environments, reinforcing the need for integrated management strategies that address ecological, veterinary, and human health dimensions in endemic conservation landscapes.

## 3 Cross-cutting insights and One Health implications

Across all thematic axes, the articles in Volume II converge on several key insights. Indirect pathogen transmission pathways, multi-host pathogens, and environmental drivers repeatedly emerge as central components of disease dynamics. Surveillance and biosecurity measures are shown to be most effective when tailored to local ecological and socio-economic contexts. Finally, intervention strategies benefit from being embedded within broader One Health frameworks that facilitate coordination among veterinary, environmental, and public health sectors.

By providing concrete examples of applied research and decision-support tools, Volume II advances the transition from conceptual understanding, as emphasized in Volume I, toward operational solutions capable of mitigating disease risks at domestic-wildlife interfaces. These contributions provide a good basis for future research and policy development aimed at mitigating transmission threats in interconnected landscapes and populations.

## 4 Conclusions

The second volume of this Research Topic consolidates and extends the contributions presented in Volume I, offering new empirical evidence, methodological developments, and management perspectives on pathogen transmission at different domestic-wildlife interfaces worldwide. The applied focus of the contributions underscores the urgency of translating eco-epidemiological knowledge generated by research into practicable, feasible strategies that protect animal health and conserve biodiversity.

Together, Volumes I and II highlight the growing complexity of domestic-wildlife interfaces worldwide and the need for integrated, evidence-based solutions grounded in One Health principles to address this ongoing and evolving challenge.
